# Probiotic Cocktail Identified by Microbial Network Analysis Inhibits Growth, Virulence Gene Expression, and Host Cell Colonization of Vancomycin-Resistant Enterococci

**DOI:** 10.3390/microorganisms8060816

**Published:** 2020-05-29

**Authors:** Wei-Sheng Sun, Yuarn-Jang Lee, Kun-Nan Tsai, Yu-Hsuan Ho, Shiuh-Bin Fang

**Affiliations:** 1 Division of Pediatric Gastroenterology and Hepatology, Department of Pediatrics, Shuang Ho Hospital, Taipei Medical University, Taipei 23561, Taiwan; adun1943@gmail.com; 2 Department of Pediatrics, School of Medicine, College of Medicine, Taipei Medical University, Taipei 110301, Taiwan; 3 Division of Infectious Disease, Department of Internal Medicine, Taipei Medical University Hospital, Taipei 110301, Taiwan; yuarn@tmuh.org.tw; 4 Department of Internal Medicine, School of Medicine, College of Medicine, Taipei Medical University, Taipei 110301, Taiwan; 5 Delta Research Center, Delta Electronics, Inc., Taipei 114501, Taiwan; brian.kn.tsai@deltaww.com (K.-N.T.); kenseifan@gmail.com (Y.-H.H.); 6 Master Program for Clinical Pharmacogenomics and Pharmacoproteomics, College of Pharmacy, Taipei Medical University, Taipei 110301, Taiwan

**Keywords:** probiotics, microbial network analysis, colonization, vancomycin-resistant enterococci, 16S rRNA sequencing

## Abstract

The prevalence of vancomycin resistant enterococcus (VRE) carrier-state has been increasing in patients of intensive care unit and it would be a public health threat. Different research groups conducted decolonizing VRE with probiotic and the results were controversial. Therefore, a systemic approach to search for the probiotic species capable of decolonizing VRE is necessary. Thus, VRE was co-cultured with ten probiotic species. The fluctuations of each bacterial population were analyzed by 16S rRNA sequencing. Microbial network analysis (MNA) was exploited to identify the most critical species in inhibiting the VRE population. The MNA-selected probiotic cocktail was then validated for its efficacy in inhibiting VRE, decolonizing VRE from Caco-2 cells via three approaches: exclusion, competition, and displacement. Finally, the expression of VRE virulence genes after co-incubation with the probiotic cocktail were analyzed with quantitative real-time PCR (qRT-PCR). The MNA-selected probiotic cocktail includes *Bacillus coagulans*, *Lactobacillus rhamnosus* GG, *Lactobacillus reuteri*, and *Lactobacillus acidophilus*. This probiotic combination significantly reduces the population of co-cultured VRE and prevents VRE from binding to Caco-2 cells by down-regulating several host-adhesion genes of VRE. Our results suggested the potential of this four-strain probiotic cocktail in clinical application for the decolonization of VRE in human gut.

## 1. Introduction

Enterococci, which are harmless commensal bacteria in the human gut [1], can cause opportunistic infections in immunocompromised individuals [2]. Enterococci acquire mobile genetic elements and develop resistance to multiple antibiotics, including the last-resort drugs vancomycin and linezolid [3,4,5]. Hospital-acquired infections that are caused by antibiotic-resistant *Enterococcus* species are difficult to treat and they can be life threatening; consequently, they have become a major public health concern and increased the economic burden of patients and governments [6,7,8]. Furthermore, *Enterococcus* species may transfer antibiotic resistance genes through conjugative plasmids to other clinically hazardous microbes [4], e.g., gram-positive *Staphylococcus aureus* [9] and gram-negative *Escherichia coli* [10]. Among hospital-acquired infections that are caused by *Enterococcus* species, *E. faecalis* and *E. faecium* account for the majority of infections, surpassing other *Enterococcus* species, including *E. casseliflavus*, *E. gallinarum*, *E. durans*, *E. avium*, *E. hirae*, *E. raffinosus*, and *E. mundtii* [11,12,13,14]. In 2017, the WHO regarded vancomycin-resistant enterococci (VRE) as requiring high priority for research and development of new antibiotics, owing to their global prevalence and the aforementioned risks to public health [15]. In addition to being the most relevant to hospital-acquired infections, *E. faecalis* and *E. faecium* are also the two main reservoirs for *vanA*, *vanB*, and *vanM*, which are the genes contributing to high levels of resistance to vancomycin; therefore, they are the main targets of studies focused on VRE. Vancomycin-resistant *E. faecalis* has been more frequently detected than other *Enterococcus* species; however, *E. faecium* is recently being increasingly detected [16,17].

Studies have indicated that probiotics might improve human health by producing metabolites that provide nutrition to human body and competing with pathogenic microorganisms [18]. To fight against pathogens, probiotic uptake provides indirect protection through immunomodulation and enhancement of the intestinal epithelial barrier [19,20,21] and direct protection through the secretion of antimicrobial molecules [22,23]; hence, the potential of probiotics in fighting against antibiotic-resistant pathogenic bacteria warrants more evaluation. *Lactobacillus* species inhibit pathogenic bacteria by producing short-chain fatty acids, hydrogen peroxide, and bacteriocins [24]. Several in vitro studies have indicated that *Lactobacillus rhamnosus* GG (*L*GG) and *L. reuteri* can antagonize VRE by reducing the attachment and colonization of VRE [25,26]. Inspired by in vitro studies, clinical trials aiming to investigate the potential of decolonizing VRE in the human gut were conducted. However, many studies did not include a reasonable cohort size, and the results were controversial. Szachta et al. reported that administering *L*GG to pediatric VRE carriers significantly reduced their VRE carrier status when compared with children in the placebo group [27]. However, administering *L*GG to adult participants with VRE carrier status in another study that was conducted by Doron et al. did not significantly reduce VRE colonization [28]. Therefore, approaches to find appropriate probiotic strains for decolonizing VRE colonization requires more studies for clarification. Studies comparing the efficacies of probiotics in a mixture and their separate strains have suggested the advantage of the probiotic mixture in improving the modulation of gut microbiota and eradicating *Helicobacter pylori* [29]. Hence, the application of probiotic mixtures to decolonize VRE in the human gut is a worthwhile attempt.

We included several probiotic species and investigated their potential of inhibiting VRE in an in vitro co-culture model to identify an effective probiotic mixture. In this study, rather than random selection of probiotic bacteria, we used a systemic approach, the microbial network analysis (MNA), which used the rule-based microbial network (RMN) algorithm [30] in order to analyze cooperative and competitive interactions among hundreds of bacterial species and infer the directions of these interactions. Thus, the optimal combination of probiotic strains for inhibiting VRE was identified. The efficacy of such a probiotic cocktail was compared with those of individual strains in the selected combination. In this study, we adopted in vitro methods, including co-culture, exclusion assay, competition assay, displacement assay, and the quantification of VRE virulence gene expression, in order to prove the potential of the probiotic cocktail that was selected by MNA in repressing the population and virulence gene expression of VRE.

## 2. Materials and Methods

### 2.1. Bacterial Strains and Culture

Table S1 lists bacterial strains, their sources, and growth conditions used in this study. VRE strains used were the clinical isolates of vancomycin-resistant *E. faecium* (VREfm) from Taipei Medical University Hospital (TMUH) and vancomycin-resistant *E. faecalis* (VREfs) from Taipei Medical University-Shang Ho Hospital (Table S1). *Bacillus coagulans* (ATCC 7050/BCRC 10606), *Bifidobacterium bifidum* (ATCC 15696/BCRC 14614), *B. longum* subsp. *infantis* (ATCC 15697/BCRC 15416), *Lactobacillus rhamnosus* GG (ATCC 53103/BCRC 16000), *Lactococcus lactis* subsp. *lactis* (CSCC 105/ BCRC 12266), *L. plantarum* subsp. *plantarum* (ATCC 14917/BCRC 10069), *Lactobacillus reuteri* (ATCC 23272/BCRC 14625), *Sporolactobacillus inulinus* (ATCC 15538/BCRC 14647), and *Streptococcus salivarius* subsp. *thermophilus* (ATCC 19258/BCRC 13869), and *Lactobacillus acidophilus* (ATCC 53544/BCRC 17009) were purchased from the Bioresource Collection and Research Center (BCRC) in Taiwan. *L. rhamnosus* GG (*L*GG^®^ Chr. Hansen, Denmark), *L. reuteri* (BioGaia^®^, Sweden), and *L. acidophilus* (Infloran^®^, Italy) were isolated from commercial products. *Bacillus coagulans* (BC1031) and *Lactobacillus acidophilus* (LA1063) were isolated from pure bacterial lyophilized powder individually purchased from Synbiotech (Taiwan). *Lactobacillus rhamnosus* GG (DSMZ 32550) and *L. reuteri* (BR101) were isolated from pure bacterial lyophilized powder that was purchased from Bio-Ray Biotech (Taiwan) individually. Anaerobiosis was achieved by incubating agar plates within an anaerobic jar (Mitsubishi Gas Chemical America, R685025, New York, NY, USA) with an anaerobic gas pack (Mitsubishi Gas Chemical America, R681001, New York, NY, USA) and incubating the liquid culture with mineral oil and 0.05% L-cysteine (Sigma-Aldrich, 168149, St. Louis, MO, USA).

### 2.2. In Vitro Bacterial Co-Culture

To fulfill the growth criteria of the ten probiotic strains and two VRE strains, they were co-cultured in MRS broth under anaerobic static condition. VREfs and VREfm were inoculated in the MRS broth with a starting OD_600_ of 0.05 and incubated with agitation one day before the co-culture with probiotics in order to establish an *Enterococcus* predominant environment for the subsequently introduced probiotic strains similar to that of human gut of VRE carrier. The next day, probiotic strains, with a density of 1.67 × 10^6^ CFU/mL each, were added to the bacterial cultures of VREfs and VREfm in MRS broth under anaerobic conditions. CFU concentrations of VREfs and VREfm were determined at fixed time points after introducing probiotic strains to evaluate the effect of the strains. The viability of the VRE population at a given time point was determined by counting the number of VRE CFUs after harvesting 100 μL of the total bacterial culture and diluting it to a proper concentration to plate out on a selective medium (CHROMagar^™^ VRE, CHROMagar, VR952, Paris, France). The experiments were independently performed in duplicate.

### 2.3. Bacterial DNA Isolation and Taxonomic Analysis

In order to isolate total bacterial genomic DNA from the in vitro co-culture, 1.5 mL of the total bacterial mixture were harvested at indicated time points and centrifuged at 5000× *g* for 5 min. The resultant bacterial pellets of two independent experiments were subjected to DNA isolation by using the bacterial genomic DNA purification kit (GeneMark, DP025, Taichung, Taiwan). The total bacterial genomic DNA samples that were obtained at individual time points were then dissected with 16S metagenomics sequencing (V3–V4 regions of 16S rRNA) to reveal the identity of individual bacterial strains and their proportion in the total population (Taipei Medical University Sequencing Core Facility). Next-generation sequencing (NGS) was performed while using the Illumina MiSeq System PE250 (San Diego, CA, USA). Raw data derived from PE250 sequencing were analyzed through the QIIME 1 pipeline [31]. Closed-reference picking and the Greengenes bacterial database [32] with 97% sequence identity were used to cluster the operational taxonomic units (OTUs) in each sample.

### 2.4. Microbial Network Analysis

The OTU tables from the QIIME analysis were exported in a tab-delimited format. Information from output tables was summarized according to taxonomy at the genus and species level. Forms were then analyzed while using the RMN algorithm [30] to infer microbial interactions that entailed competitive and cooperative relationships between VRE and probiotics. All of the parameters from the RMN pipeline followed original settings, except for the cut-off D value, which was set as 1. In addition, some probiotics that had cooperative relationships with the VRE or competitive relationships with other probiotics were not considered.

### 2.5. RNA Isolation and Quantitative Real-Time PCR

Before the quantification of mRNA expression by using qRT-PCR, RNA was isolated using the total RNA purification kit (GeneMark, TR01, Taichung, Taiwan) from bacterial pellets after centrifugation of bacterial co-cultures, according to manufacturer’s instructions. After extraction, the concentrations of RNA samples were determined using DeNovix DS-11+Spectrometer (DeNovix, Wilmington, DE, USA). After determining the quality and concentration of RNA samples by measuring the OD_260/280_, 1 μg of RNA was treated with DNase I (AMPD1, Sigma-Aldrich, St. Louis, MO, USA) to remove any contaminated chromosomal DNA and then reverse-transcribed into cDNA using the iScript™ cDNA Synthesis Kit (Bio-Rad, 1708891, Hercules, CA, USA). Quantitative real-time PCR was then performed by applying synthesized cDNA samples, corresponding primer pairs (Table S2), and SYBR^®^ Green Master Mix (Bio-Rad, 1708886, Hercules, CA, USA) in triplicate to the CFX96 Real-Time System (Bio-Rad, Hercules, CA, USA). The comparative Ct (^ΔΔ^Ct) system was used to determine the relative mRNA expressions of selected VREfm virulence genes. The acquired mRNA expressions were normalized to the level of an internal control (16S rRNA of *E. faecium*) of each sample; subsequently, the fold changes against the corresponding control groups were calculated and expressed as log_2_ fold change.

### 2.6. Caco-2 Cell Culture

The Caco-2 cell line was purchased from the Bioresource Collection and Research Centre Taiwan (BCRC No. 67001, originally from [ATCC No. HTB-37] and routinely cultured in Dulbecco’s modified Eagle’s minimal essential medium (DMEM) (12800-017, Gibco, ThermoFisher Scientific, Waltham, MA, USA) with 10% fetal bovine serum (10437-028, Gibco, ThermoFisher Scientific, Waltham, MA, USA) at 37 °C and under 5% CO_2_; media were changed every two to three days. For in vitro VREfm adherence assays, the Caco-2 cells were seeded in 12-well cell culture plates (150628, Nunc, Waltham, MA, USA) at a density of 0.1 × 10^6^ cells/well and maintained by replacing the media every two to three days to reach a complete confluency at 1 × 10^6^ cells/well after 10 days.

### 2.7. VREfm Adherence to Caco-2 Cells In Vitro

The adherence of VREfm to Caco-2 cells was inhibited using probiotics according to a previous procedure, with modifications [26]. For the exclusion assay, Caco-2 cells in each well of 12-well plates forming confluent monolayers were washed twice with Dulbecco’s phosphate buffer saline (DPBS) (D5652, Sigma-Aldrich, St. Louis, MO, USA) and cultured with probiotic strains (approximately 3.33 × 10^8^ CFU/mL) for 1 h. After incubation with probiotics, the Caco-2 cells were washed with DPBS twice and incubated with VREfm (approximately 3.33 × 10^6^ CFU/mL) for another 1 h. Subsequently, the Caco-2 cells were washed twice with DPBS and then incubated with 0.05% Triton-X100 (T8787, Sigma-Aldrich, St. Louis, MO, USA) at 37 °C for 20 min. The adherent VREfm were then harvested with Caco-2 cell lysates and plated out on selective media after appropriate serial dilutions. For the displacement assay, the Caco-2 cells were initially cultured with VREfm for 1 h and then incubated with probiotics for another 1 h. The rest of the procedures remained the same as in the exclusion assay. For the competition assay, Caco-2 cells were initially washed twice with DPBS and then co-incubated with probiotic strains (approximately 3.33 × 10^8^ CFU/mL) and VREfm (approximately 3.33 × 10^6^ CFU/mL) for 2 h. After co-incubation, Caco-2 cells were washed twice to remove unattached bacterial cells and then subjected to the same cell lysis and plating out procedures. VREfm and probiotics of appropriate bacterial cell numbers were all resuspended in serum-free DMEM after determining their CFU concentrations before incubation with Caco-2 cells. The efficacies of probiotic treatment were compared with those of the control group without probiotics for the CFU number of VREfm remained attached to Caco-2 cells after assays. The experiments were independently performed in triplicate.

### 2.8. VREfm and Probiotics Co-Cultured with Caco-2 Cells

The Caco-2 cells were seeded in T75 cell culture flasks (156499, Nunc, ThermoFisher Scientific, Waltham, MA, USA) in advance at a cell density of 1.7 × 10^6^ cells/flask and cultured for 10 days to reach confluency at 1.7 × 10^7^ cells/flask. The CFU concentrations of VREfm and probiotic strain (*B. coagulans*_BC1031, *L. rhamnosus* GG_DSMZ 32250, and *L. reuteri*_BCRC BR101, and *L. acidophilus_*LA1063) inocula prepared one day in advance were determined. The inocula of VREfm and four probiotic strains were collected for an MOI of 350 to Caco-2 cells and washed twice with DPBS and then resuspended with serum-free DMEM. Caco-2 cells in the T75 flask were washed twice with DPBS and then co-incubated with VREfm and the probiotic combination for 2.5 h at 37 °C under 5% CO_2_. Bacterial cells of VREfm in supernatants were then harvested and washed twice with DPBS before RNA isolation. The same amount of VREfm in the absence of probiotic combination was incubated with Caco-2 cells in a T75 flask in parallel and collected as a control group. The same amount of VREfm that was incubated under the same culture condition without contacting Caco-2 cells was harvested as another control group.

### 2.9. Statistical Analysis

All of the data are presented as means and analyzed while using GraphPad Prism (version 5.0) (GraphPad Software Inc., La Jolla, CA, USA). Student’s *t*-test was used to determine significant differences between the experimental groups; different *p* values between paired data indicated the following levels of significance. *p* < 0.05, < 0.01, and < 0.001 indicated statistically significant, highly significant, and extremely significant differences, respectively (* *p* < 0.05, ** *p* < 0.01, and *** *p* < 0.001).

## 3. Results

### 3.1. Probiotic Mixture Significantly Reduced VRE Number in an In Vitro Co-Culture

**Figure 1:**
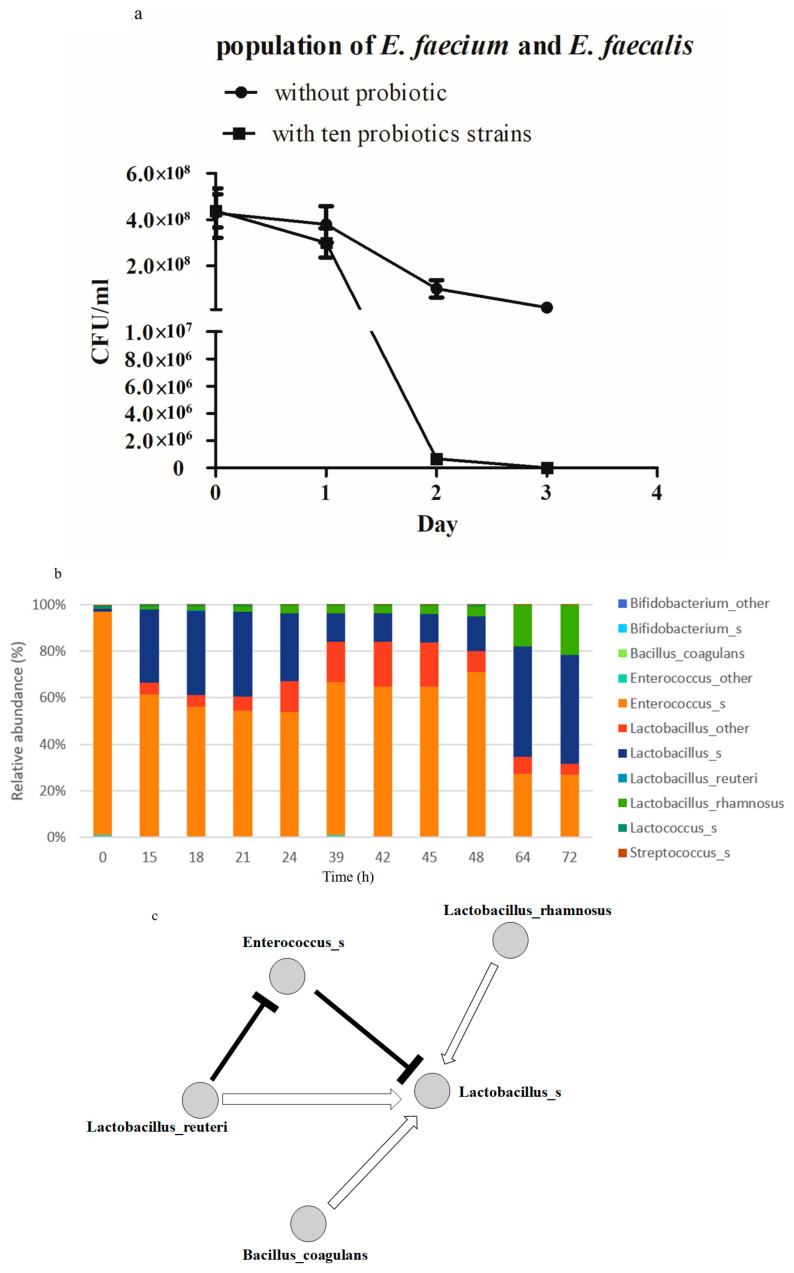
Probiotic mixture enhanced reduction of vancomycin-resistant enterococci (VRE) CFU in in vitro culture. Overnight-incubated VRE (*E. faecalis* and *E. faecium*) in the MRS broth were supplemented with or without a mixture of ten probiotic strains under anaerobic conditions for three days. (**a**) CFU concentrations of *E. faecalis* and *E. faecium* in co-culture mixture were determined daily and compared between the ten-probiotic co-culture group and control group without probiotic. (**b**) The compositions of the total bacterial population at 11 time points after co-culture revealed by 16S rRNA sequencing: each bacterial strain has been allotted a different-colored block in bar charts, with size that is proportional to its percentage in the total population at a given time point. Note that, as the sequence of the V3–V4 region of 16S rRNA may have been insufficient to distinguish bacterial species of the same genus, some of the bacterial species were numerated with only genus. In addition, one of the probiotic strains, namely *S. inulinus*, was not detected. (**c**) Microbial network analysis conducted based on the results of 16S rRNA sequencing results to identify dominant probiotic strains during co-culture with VRE. Closed circles represent different bacterial species in the VRE-probiotic co-culture. Black-mallet-head lines indicate competitive relationships (mallet head pointed to the species being competed), whereas white arrows indicate cooperative relationships. Experiments of bacterial co-culture and its derived 16S rRNA sequencing analysis were independently performed in duplicate.

Ten strains of probiotic bacteria belonging to the genera of *Bacillus*, *Bifidobacterium*, *Lactobacillus*, *Lactococcus*, *Sporolactobacillus*, and *Streptococcus* were included in this study to determine a formula of probiotics to decolonize VRE in the human gut, as follows: *Bacillus coagulans*, *Bifidobacterium bifidum*, *Bifidobacterium longum* subsp. *infantis*, *Lactococcus lactis* subsp. *lactis*, *Lactobacillus plantarum* subsp. *plantarum*, *Sporolactobacillus inulinus*, *Streptococcus salivarius* subsp. *thermophilus*, *Lactobacillus rhamnosus* GG, *Lactobacillus reuteri*, and *Lactobacillus acidophilus*. The phenotypes of of VRE strains in vancomycin resistance were confirmed by disk diffusion assay and E-TEST following the guideline of CSLI (Table S3) in advance. *van A* was also detected in both strains with the gene-specific primer pair (Figure S1). The potential of inhibiting VRE was then investigated by co-culturing the mixture of ten probiotic strains with the inocula containing both VREfs and VREfm. The mixture of the ten probiotic strains expedited the decline of VREfs and VREfm populations, when compared with that observed in the group without probiotics, according to results shown in Figure 1a. The total bacterial pellets of 11 time points within three days of VRE-probiotic co-cultures were collected for genomic DNA isolation to identify the most critical probiotic strains and downsize the formula for outcompeting VRE. The fluctuations of bacterial populations during co-culture were detected through NGS, which revealed the V3–V4 region sequences of bacterial 16S rRNA and therefore the identities of all bacterial strains and their relative abundances. VRE constituted over 90% of the total population at the beginning of the co-culture, which decreased to approximately 60% between 15 and 48 h after co-culture and further declined to 30% after 64 h. By contrast, *L. rhamnosus*, *Lactobacillus_*S, and *Lactobacillus_*other dominated at the end of co-culture (Figure 1b). The results of NGS were initially analyzed with the QIIME 1 pipeline and then clustered for OTUs in each sample. The constructed OTUs were then subjected to MNA based on a special algorithm, the RMN algorithm, which suggested the directions of bacterial competitive and cooperative relationships, helping to predict the most critical species during the co-culture and interactions between them. According to the MNA results, *B. coagulans*, *L. reuteri*, *L. rhamnosus* GG, and another *Lactobacillus* species were critical for suppressing the population of VRE, among which *L. reuteri* played the most important role (Figure 1c).

**Figure 2:**
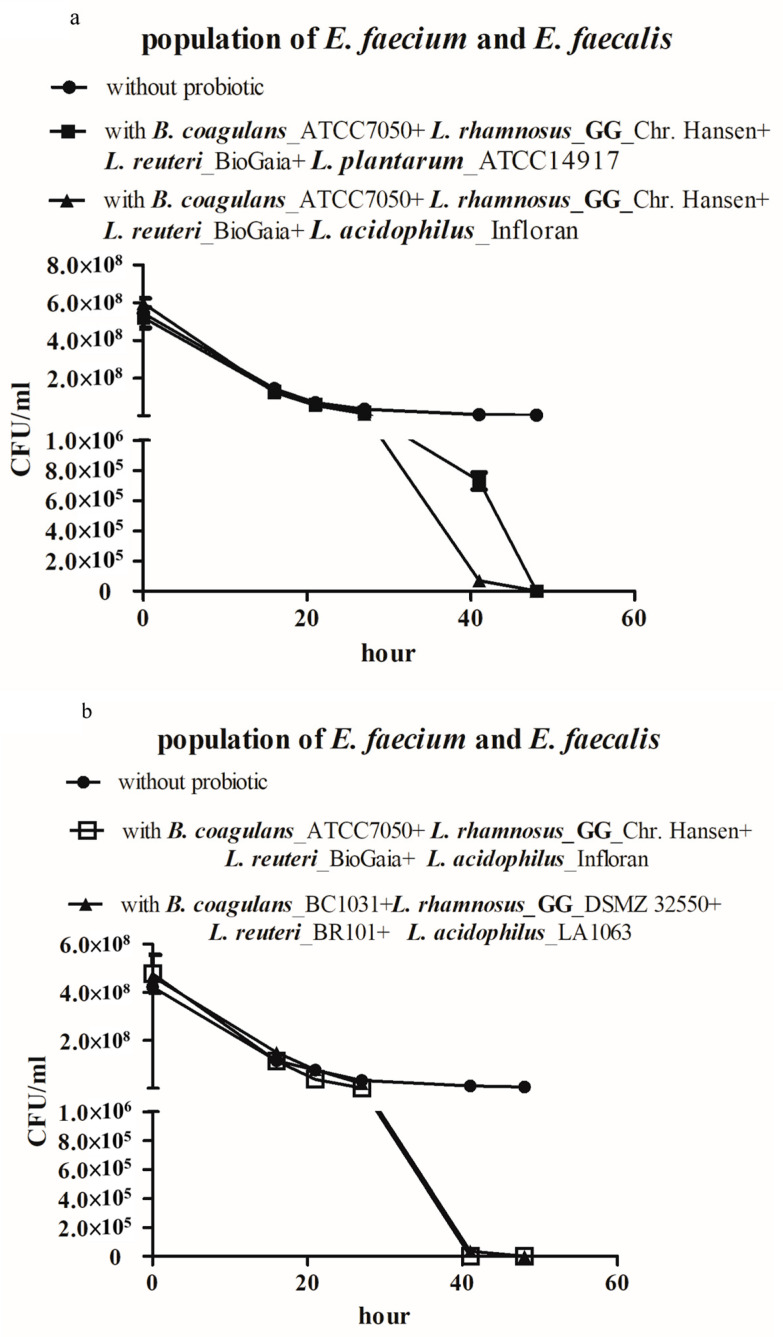
Microbial network analysis (MNA)-assisted identification of critical species for inhibiting VRE from the probiotic mixture. (**a**) The efficacies of *L. plantarum*_ATCC14917 and *L. acidophilus*_Infloran in repressing the co-cultured VRE population when in combination with *B. coagulans*_ATCC7050, *L. rhamnosus* GG_Chr. Hansen, and *L. reuteri*_BioGaia were compared. (**b**) Efficacy of suppressing co-cultured VRE population was compared between a combination of probiotic strains *B. coagulans*_ATCC7050, *L. rhamnosus* GG_Chr. Hansen, *L. reuteri*_BioGaia, and *L. acidophilus*_Infloran and the combination substitutes of the four probiotic strains, BC1031, DSMZ 32550, BR101, and LA1063.

The efficacy of these two strains against the VRE population in co-cultures was compared in combination with *B. coagulans*, *L. rhamnosus* GG, and *L. reuteri* to determine whether the unknown *Lactobacillus* species was *L. plantarum* or *L. acidophilus*. The combination of *B. coagulans*, *L. rhamnosus* GG, *L. reuteri*, and *L. acidophilus* was more effective than the combination of *B. coagulans*, *L. rhamnosus* GG, *L. reuteri*, and *L. plantarum* in reducing VRE concentrations, according to the results of co-culturing (Figure 2a). This suggests that *L. acidophilus* was a more promising component than *L. plantarum* in our probiotic formula.

In order to avoid the possibility of patent infringement, similar strains from alternative sources were adopted, as the three *Lactobacillus* species in our formula were isolated from commercial products (Table S1). ATCC originated-*Lactobacillus rhamnosus* GG (ATCC 53103/BCRC 16000), *Lactobacillus reuteri* (ATCC 23272/BCRC 14625), and *Lactobacillus acidophilus* (ATCC 53544/BCRC 17009) were purchased from BCRC. Samples of *B. coagulans_*BC1031 and *L. acidophilus_*LA1063 were purchased from SynBiotech (Taiwan), whereas samples of *L. rhamnosus* GG_DSMZ 32,250 and *L. reuteri_*BCRC BR101 were purchased from Bio-Ray Biotech (Taiwan). The efficacies of the combination of the original four strains and the combination of their alternatives were then compared in VRE-probiotic co-cultures. *B. coagulans_*BC1031 in combination with *Lactobacillus rhamnosus* GG (ATCC 53103), *Lactobacillus reuteri* (ATCC 23272), *Lactobacillus acidophilus* (ATCC 53544) showed similar but relative weak activity in suppressing the co-cultured VRE when comparing to the original combination (Figure S2). The combination of *B. coagulans_*BC1031, *L. rhamnosus*_GG_DSMZ 32250, *L. reuteri*_BR101, and *L. acidophilus_*LA1063 showed effects that were comparable to those of the original combination with regard to reduction of VRE (Figure 2b). This result indicates that *B. coagulans_*BC1031 and *L. rhamnosus* GG_DSMZ 32250, *L. reuteri_*BCRC BR101, and *L. acidophilus_*LA1063 can be used as substitutes for original strains in further studies.

### 3.2. MNA-Selected Four-Probiotic Mixture Reduced the Number of Co-Cultured VRE More Significantly than Did Individual Strains

**Figure 3:**
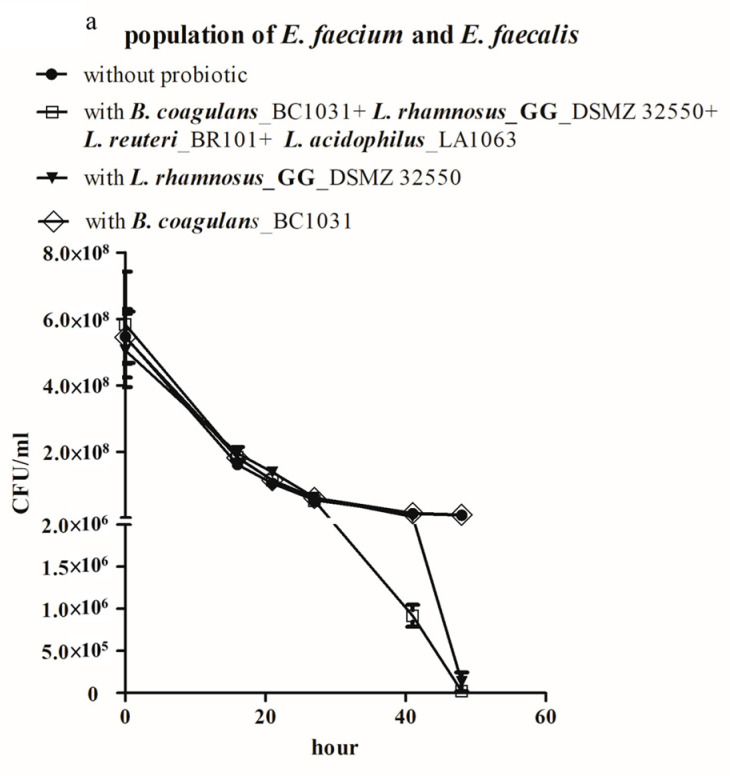

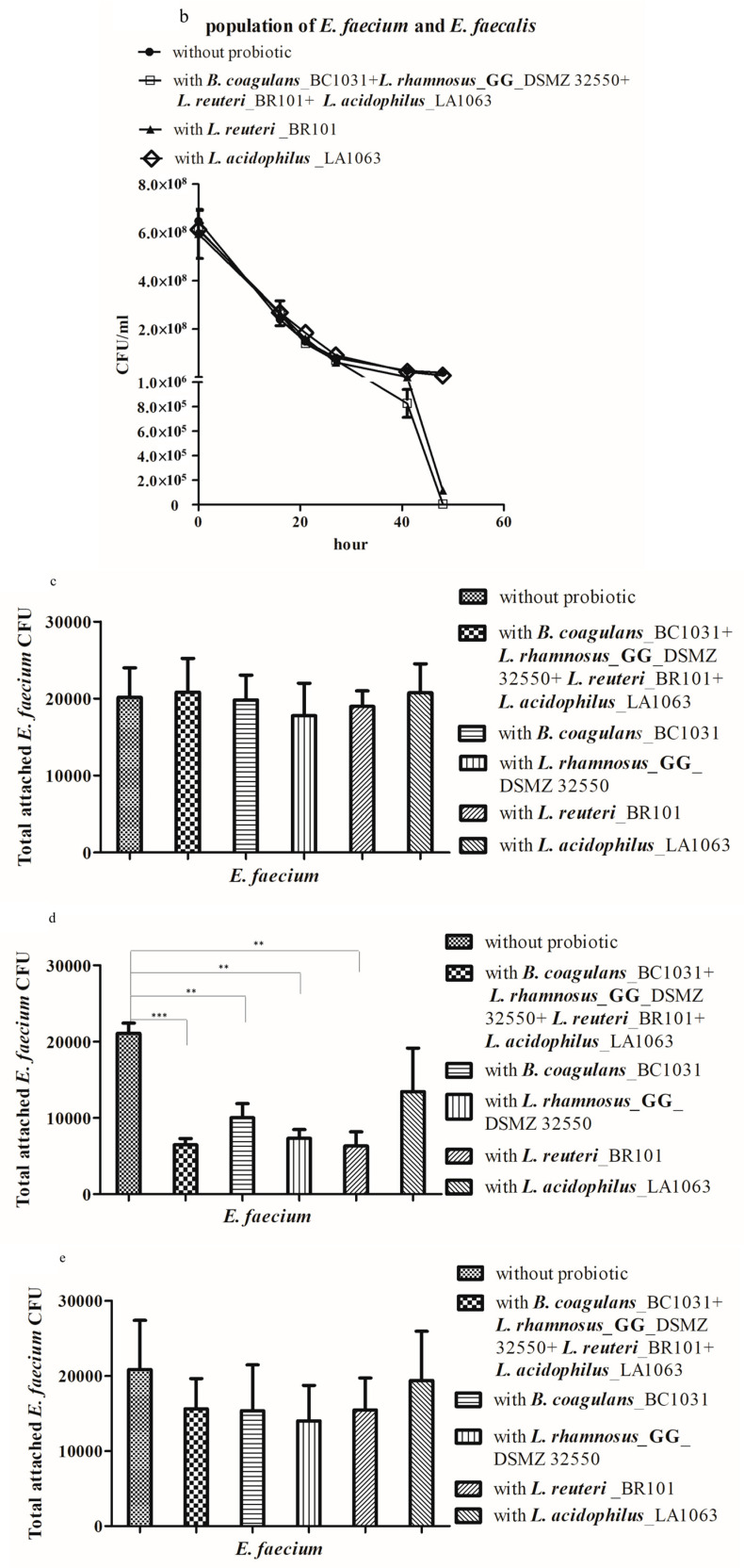
The combination of four probiotic strains showed better efficacy than that of individual strains against co-cultured VRE. Individual strains of (**a**) *B. coagulans_* BC1031 or *L. rhamnosus* GG_ DSMZ 32550, (**b**) *L. reuteri_* BR101 or *L. acidophilus_* LA1063 were compared with the combination of the four strains for the efficacy against the co-cultured VREfs and VREfm population. (**c**) Efficacies of pre-incubated individual probiotics strains and that of the combination of four probiotic strains were compared with exclusion assay. (**d**) Competition assay was performed to compare the ability of individual probiotics strains and combination of the four probiotic strains in competing with *E. faecium* for binding to Caco-2 cells. (**e**) Comparison of the efficacies of individual strains and the combination of the four strains in displacing *E. faecium* adherent to Caco-2 cells. The probiotic combination used here consisted of the four strains in equal ratio. The experiments of bacterial co-cultures were independently performed in duplicate, whereas the experiments of VRE adherence to Caco-2 cells were independently performed in triplicate. <0.01, and <0.001 indicated statistically significant, highly significant, and extremely significant differences, respectively and depicted as ** *p* < 0.01, and *** *p* < 0.001 in figures.

The efficacies of the mixture of the four probiotic strains in an equal ratio and individual strains were compared to further confirm the necessity of combining the four probiotic strains. In the VRE-probiotic co-culture model, a combination of *B. coagulans_*BC1031, *L. rhamnosus* GG_DSMZ 32250, *L. reuteri_*BCRC BR101, and *L. acidophilus_*LA1063 (Figure 3a, squares) surpassed *B. coagulans_*BC1031 (Figure 3a, diamonds) or *L. rhamnosus* GG_DSMZ 32,250 (Figure 3a, inverted triangles) alone in reducing VREfm and VREfs populations and showed a significant difference at the time point of 41 h. Supplementing *L. rhamnosus* GG_DSMZ 32,250 alone did not significantly reduce the number of VRE until at the time point of 48 h (Figure 3a, inverted triangles), whereas the VRE population in the group that was supplemented with *B. coagulans_*BC1031 alone (Figure 3a, diamonds) did not show any difference from the VRE only group (Figure 3a, circles). The efficacies of *L. reuteri_*BCRC BR101 and *L. acidophilus_*LA1063 alone were also evaluated. *L. reuteri_*BCRC BR101 showed a relatively weaker efficacy than that of the combination of the four strains at 41 and 48 h (Figure 3b, squares vs. triangles), whereas *L. acidophilus_*LA1063 slightly enhanced the reduction of co-cultured VRE, but did not significantly diminish the majority of VRE (Figure 3b, diamonds).

Caco-2 cells, a well-established human epithelial colorectal adenocarcinoma cell line, have been widely utilized as an in vitro model in studying adherence of pathogenic or probiotic bacterial species to gut epithelial cells [26,33]. The efficacy of reducing VRE adherence to Caco-2 cells was compared between the four-strain probiotic combination and individual strains because *L. reuteri* reduces the adherence of pathogenic bacteria to Caco-2 cells [26]. Three different approaches were adopted to evaluate the effectiveness of probiotics in reducing the adherence of VRE. In the exclusion assay, probiotics were incubated with Caco-2 cells in advance and their effectiveness in blocking the attachment of VRE that was introduced later was evaluated; in the competition assay, probiotics were simultaneously supplemented with VRE and their efficacy of competing with VRE in adherence to Caco-2 cells was evaluated; and in the displacement assay, probiotics were introduced to Caco-2 cells that were pre-incubated with VRE and their effectiveness in displacing the attached VRE was evaluated [26]. All of the probiotic-introducing treatment would be compared with its counterpart control that without probiotic. We focused on VREfm, which was reported to show more severe antibiotic resistance, to simplify our study [34]. Supplementing probiotics as individual strains or in combination did not significantly prevent the attachment of the later introduced VREfm to Caco-2 cells, according to the results shown in Figure 3c. By contrast, when probiotics, except for *L. acidophilus*_LA1063, were supplemented along with VREfm, the attached VREfm of each group was reduced to 30–50% (Figure 3d). When probiotics were administered to Caco-2 cells pre-incubated with VREfm, the remaining attached VREfm reduced to 65–77% of that observed in the control group without probiotics, except for the one that was supplemented with *L. acidophilus_*LA1063 (Figure 3e). Although the combination of the four probiotic strains did not show significantly better efficacy than that of individual strains of *B. coagulans_*BC1031, *L. rhamnosus* GG_DSMZ 32250, or *L. reuteri_*BCRC BR101 in reducing the adherence of *E. faecium* to Caco-2 cells, it was more promising in reducing the VRE concentrations in co-cultures and, therefore, more suitable for subsequent studies.

### 3.3. The Four-Probiotic Mixture Significantly Downregulated the Expression of Nine Virulence Genes of VREfm

**Figure 4:**
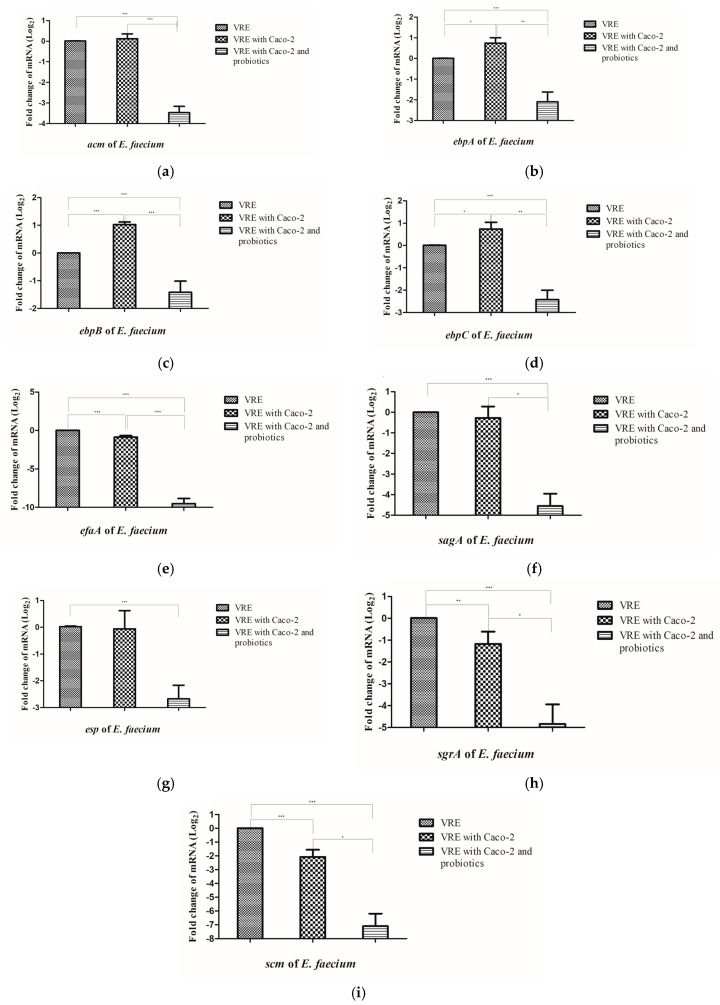
Probiotic combination reduced the expression of VRE *E. faecium* virulence genes when co-incubated with Caco-2 cells. The mRNA expression levels of *E. faecium* virulence genes: (**a**) *acm*, (**b**) *ebpA*, (**c**) *ebpB*, (**d**) *ebpC*, (**e**) *efaA*, (**f**) *sagA*, (**g**) *esp*, (**h**) *sgrA*, and (**i**) *scm* were evaluated after incubation in serum-free Dulbecco’s modified Eagle‘s minimal essential medium (DMEM) alone (VRE), with Caco-2 cells (VRE and Caco-2), and with Caco-2 cells and probiotics (VRE with Caco-2 and probiotics) at 37 °C under 5% CO_2_ for 2.5 h. The experiments were independently performed in triplicate. *p* < 0.05, < 0.01, and < 0.001 indicated statistically significant, highly significant, and extremely significant differences, respectively and depicted as * *p* < 0.05, ** *p* < 0.01, and *** *p* < 0.001 in figures.

The probiotic combination showed significant efficacy in competing with VREfm for binding to Caco-2 cells. This implies that virulence genes that are involved in bacterial adherence to host cells might be affected during interactions between VREfm and probiotics. The expression of VREfm virulence genes in the presence of host cells and interaction with probiotics were evaluated by co-culturing VREfm and the four-strain probiotic mixture in equal ratios with Caco-2 cells for 2.5 h in order to validate this hypothesis. The bacterial cells of VREfm were then harvested for quantifying the expressions of virulence genes associated with adherence to host cells. VREfm co-cultured with both Caco-2 cells and probiotics was compared with VREfm co-cultured with only Caco-2 cells and with VREfm not contacting Caco-2 cells in the expression levels of the selected nine virulence genes. Nine adherence-related genes were included in this study. *acm* encodes the adhesin to facilitate the binding of type I and IV collagen [35]. *ebpA*, *ebpB*, and *ebpC* are the genes encoding for subunits of *E. faecium* pilus that are required for fibrinogen binding [36,37]. EfaA shares 42–63% similarity of amino acid sequences with a family of streptococcal virulence and adhesion proteins [38]. SagA is capable of adhering to a wide range of extracellular proteins [39]. Esp, which is an important antigenic marker of endocarditis, is required for biofilm formation [40]. *sgrA* encodes an adhesion protein with the LPXTG motif and it is potentially required for biofilm formation [41]. *scm* encodes a collagen-binding protein [37]. The results of our study demonstrated that *acm*, *sagA*, and *esp* exhibited similar mRNA expressions after contacting Caco-2 cells (Figure 4a,f,g). The mRNA expressions of *ebpA*, *ebpB*, and *ebpC* slightly increased to approximately two-fold (Figure 4b–d). Meanwhile, the mRNA expressions of *efaA*, *sgrA*, and *scm* decreased two- to four-fold after contacting Caco-2 cells (Figure 4e,h,i). In addition, all of the nine virulence genes of VREfm were significantly downregulated after co-culturing with Caco-2 cells in the presence of the four-strain probiotic mixture. These results suggest that adherence-associated genes that are responsible for different host environments may act differently under a given condition, and their mRNA expressions were suppressed when VREfm interacting with the four-strain probiotic mixture, thus reducing the adherence of VREfm to Caco-2 cells.

## 4. Discussion

Discovery of alternative antibacterial drugs and therapies is warranted owing to the emergence of multiple-drug-resistant bacteria and the shortage of new antibiotics. The cooperation and competition between microorganisms can be exploited to develop main or supportive therapies [42,43]. We used MNA driven by the RMN algorithm and found that the combination of *B. coagulans*, *L. rhamnosus* GG, *L. reuteri*, and *L. acidophilus* significantly repressed the co-cultured VRE population, reduced the attachment of VREfm to Caco-2 cells, and downregulated the mRNA expression of VREfm virulence genes. To the best of our knowledge, this is the first study in which multiple probiotic species were used in order to prevent the colonization of VRE in the human intestinal epithelial cell line, suggesting the feasibility of decolonizing VRE in the human gut.

A study reported that a combination of probiotic species decolonized extended spectrum beta-lactamase-producing *Enterobacteriaceae* in a randomized clinical trial, [44] and led to better outcomes than those that were yielded by single species in clinical trials of modulating gut microflora and eradicating *H. pylori* infection [29]. This might be attributed to the mutualistic or commensalistic relationships between probiotic species [45]. All probiotic species in the four-probiotic mixture have been reported to possess bioactivities that help fight against VRE; administering *B. coagulans* reduced the colonization of VRE in a mouse model [46]; supplementing *L. rhamnosus* GG significantly decolonized VRE in participants in two independent clinical trials [27,47]; *L. reuteri* was effective in reducing the attachment of VRE to Caco-2 cells [26]; and, *L. acidophilus* enhanced the host immune response of *C. elegans* against *E. faecalis* infection [48]. In our study, these four probiotic strains showed relatively weak or no efficacies in reducing the co-cultured VRE population individually, when comparing with the efficacy of their combination. This might be attributed to the synergistic effect of cooperative relationships among these four strains (Figure 3a,b), as suggested by MNA based on the RMN algorithm in this study. The RMN algorithm is a rule-based microbial network algorithm that not only suggests cooperative and competitive relationships between microorganisms, but also infers the directions of such relationships; therefore, the algorithm helps to suggest the most pivotal species by reconstructing regulatory networks in a complex microbial community [30]. In addition, RMN proved beneficial in dissecting the interactions among neutrophilic iron-oxidizing Zetaproteobacteria and constructing microbial networks in the hydrothermal vent ecosystems [49]. MNA was useful in identifying the most critical probiotic strains among the ten selected probiotic strains. Given that the NGS data of the V3–V4 region of total bacterial 16S rRNA failed to reveal the specific species of all *Lactobacillus* strains, the MNA still suggested that *L. reuteri* and *L. rhamnosus* GG play important roles; this helped to reduce the time that is required for confirmation. After switching to alternative sources of the four probiotic strains (Figure 2b and Figure S2), the alternative four strains still generated similar results, which suggested that the effect of these four strains on VRE is not specific to the strains from specific sources and consolidates the choice of these four strains.

Antibiotic treatment to VRE carriage is hampered by the intrinsic resistance of enterococci, which is contributed by a hardened cell wall and the accumulation of antibiotic resistance genes, and might facilitate VRE overgrowth and resistance gene transmission [50]. Thus, oral administration of probiotics is an alternative that was evaluated in several studies. In trials which were conducted by Manley et al. and Szachta et al., administration of *L. rhamnosus* GG significantly reduced VRE colonization in trial participants [27,47]. However, a similar approach did not yield positive results in the trial that was conducted by Doron et al. [28]. Lcr35, an *L. rhamnosus* probiotic strain phylogenically close to *L*GG [51], did not have a significant effect on VRE carriage [52]. These controversial results may be attributed to different operation protocols (e.g., probiotic strains, dosage, and duration) and cohort size, as well as to the gut microbiome diversity between the cohorts of different trials, which could profoundly influence the colonization of probiotic species [53]. The strategy of administering multiple probiotic species, including *L. rhamnosus* and *L. acidophilus*, was adopted in another trial; however, it failed to prevent the colonization of ampicillin-resistant *E. faecium* [54], which suggested that MNA-assisted, in vitro study-supported selection of a mixture of multiple probiotics can be more promising in clinical trials. Fecal microbiota transplantation (FMT) to decolonize VRE can also be regarded as a multiple-species treatment that reduced the colonization of VRE in a mouse model and a clinical trial [55,56]. However, FMT treatment is more invasive and it carries a risk of multiple-drug-resistant pathogenic infections. Therefore, the administration of probiotics would be a safer option than FMT for VRE-carrying patients with other underlying diseases or compromised immune system.

Adhesion molecules are important for the colonization and dissemination of enterococci in the host environment [57]. In our study, decreased colonization by VRE in Caco-2 cells was observed under treatment with the four-probiotic mixture, accompanied by the downregulation of adherence-associated genes. Nine adherence-associated genes that were included in this study exhibited differential responses after incubation with Caco-2 cells and they were significantly downregulated when the four-strain probiotic mixture was incubated with VREfm (Figure 4a–i). A similar trend in the downregulation of these nine genes might be a coordinated global regulation. In *E. faecalis*, the homologous genes of *ebpA*, *ebpB*, *ebpC*, and *efaA* were reported to be regulated by FsrABC [58,59], a quorum-sensing system that regulates various virulence genes [60]. FsrABC shares sequence homology with the *Staphylococcus aureus* AgrABC system [60], which can be inhibited by fengycins that are produced by *Bacillus* species [23]. Therefore, the likelihood of FsrABC, the AgrABC-like quorum-sensing system in *E. faecalis* and *E. faecium*, being regulated by oligopeptides or other secretory molecules produced by the four-strain probiotic mixture and downregulating the expressions of various adhesion genes is worth investigating. Whether all of the nine adherence-associated genes are under regulation of FsrABC warrants further investigation. In addition to the downregulation of VRE adherence-associated genes, other underlying mechanisms may also contribute to the reduced attachment of VRE to Caco-2 cells. *L*GG possesses a pilus gene cluster that shares sequence similarity with the pilus gene cluster of VRE; therefore, preventing VRE from attaching to mucus by direct competition [25]. Bacteriocin produced by probiotic species contributes to the reduction of VRE colonization [61]. In addition, *Lactobacillus* species induced the expression of antimicrobial peptide human LL-37 and reduced VRE persistence in mice [62]. Whether the four-probiotic mixture adopts similar mechanisms remains unclear.

## 5. Conclusions

In conclusion, we introduced an MNA-selected four-probiotic mixture that was effective in inhibiting co-cultured VRE and reducing the attachment of VRE to Caco-2 cells in vitro. The mixture is being used in the first clinical trial that aims to decolonize VRE in the human gut by using multiple probiotic species in order to validate the efficacy of this four-probiotic mixture in the human gut (ClinicalTrial.gov ID: NCT03822819). The mechanism of action of this four-probiotic mixture should be investigated in additional prospective studies, and these studies can provide clues for potential optimization of this mixture in the future.

## Supplementary Materials

The following are available online at https://www.mdpi.com/2076-2607/8/6/816/s1, Figure S1: *vanA* detection in VREfs and VREfm of this study, Figure S2: Efficacy comparison between the four-strain probiotic combination of different sources, Table S1: Strains used in this study and their culture conditions, Table S2: Primers used in this study, Table S3: Disk diffusion assay result of VRE strains in this study.
