# Supplementary figures and images for: Probiotic Cocktail Identified by Microbial Network Analysis Inhibits Growth, Virulence Gene Expression, and Host Cell Colonization of Vancomycin-Resistant Enterococci
Source: Microorganisms. 2020 May 29;8(6):816. doi: 10.3390/microorganisms8060816 (PMC7357164; doi:10.3390/microorganisms8060816)

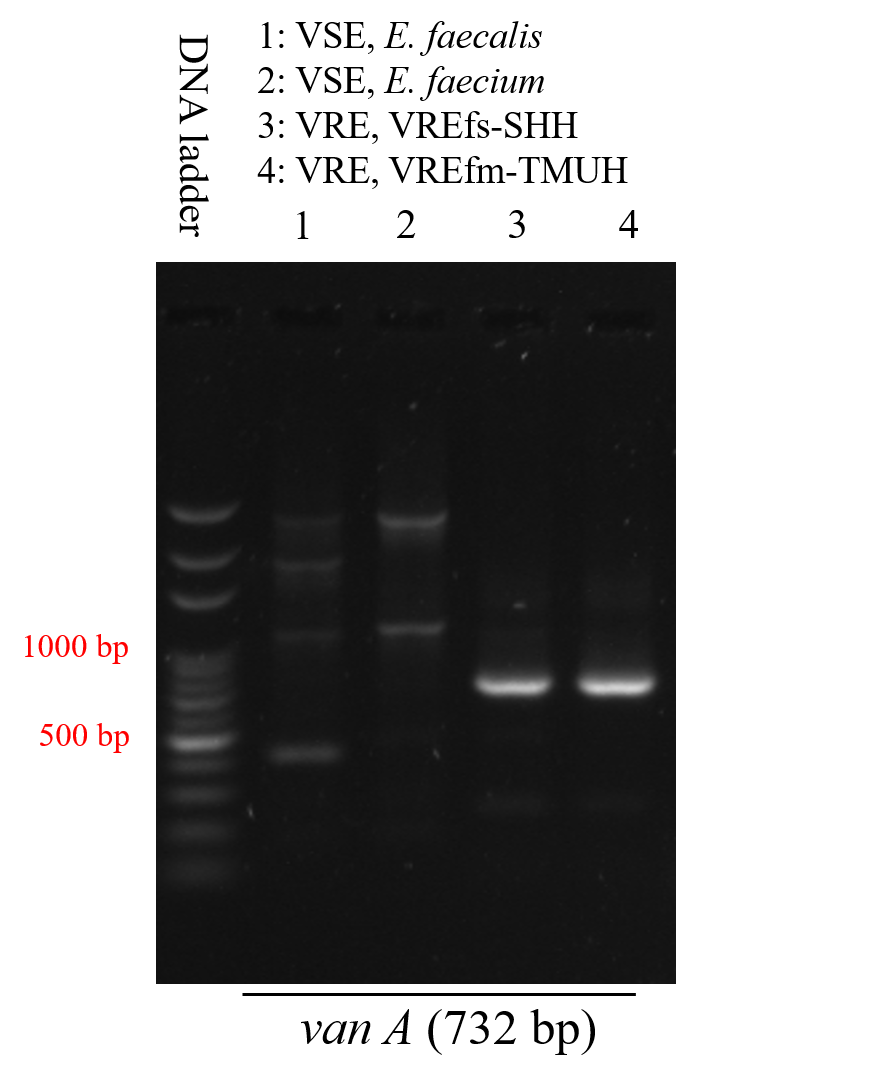

Supplement: Supplementary file 1 [file microorganisms-08-00816-s001.zip › Supplementary/Figure S1.tif]

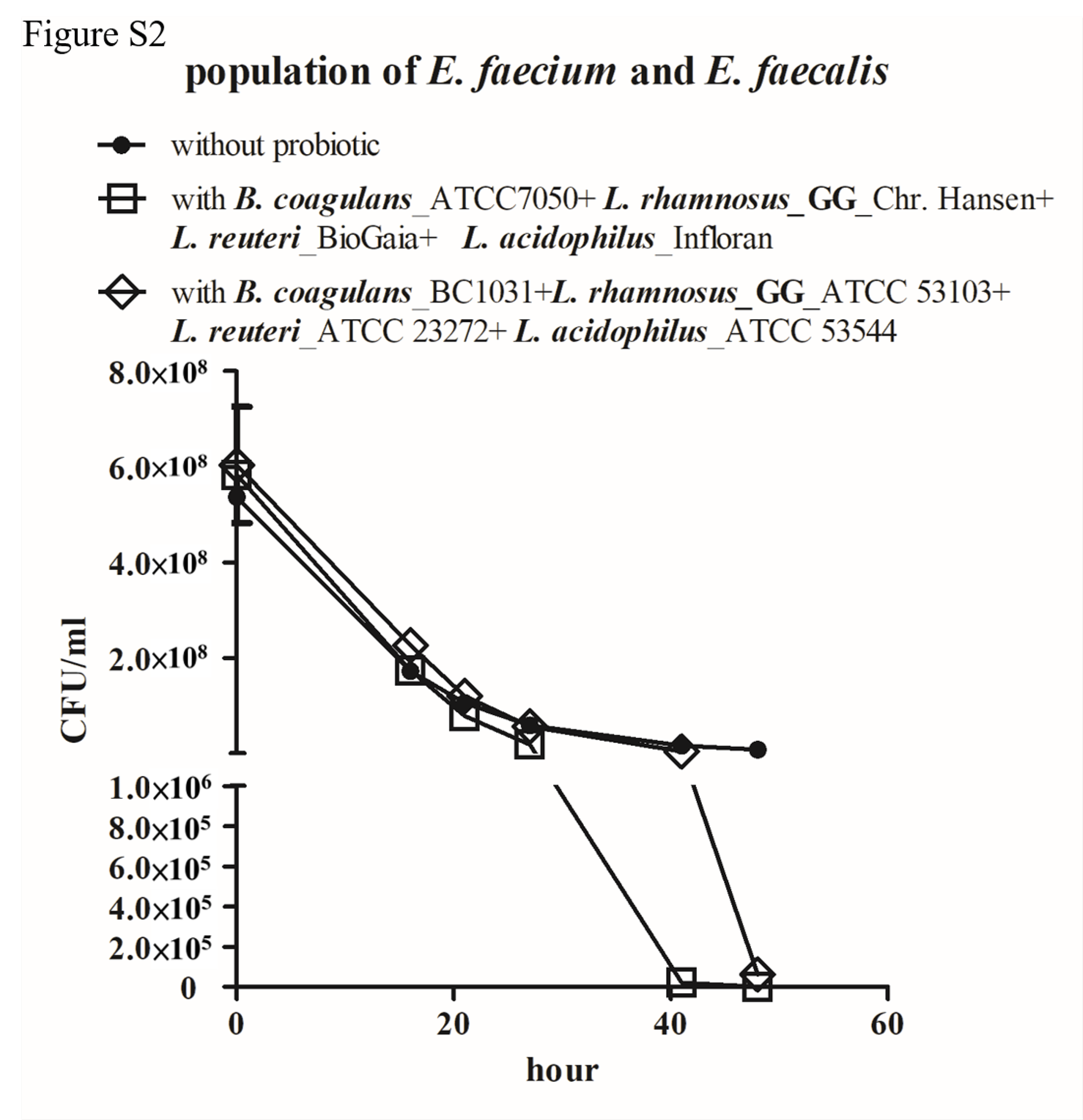

Supplement: Supplementary file 1 [file microorganisms-08-00816-s001.zip › Supplementary/Figure S2.tif]
